# Analysis of Ergot Alkaloids

**DOI:** 10.3390/toxins7062024

**Published:** 2015-06-03

**Authors:** Colin Crews

**Affiliations:** Fera Science Ltd., Sand Hutton, North Yorks YO41 1LZ, UK; E-Mail: colin.crews@fera.co.uk; Tel.: +44-1904-462-549; Fax: +44-1904-462-111

**Keywords:** ergot, LSD, ergotamine, lysergic acid, ergovaline, mycotoxins

## Abstract

The principles and application of established and newer methods for the quantitative and semi-quantitative determination of ergot alkaloids in food, feed, plant materials and animal tissues are reviewed. The techniques of sampling, extraction, clean-up, detection, quantification and validation are described. The major procedures for ergot alkaloid analysis comprise liquid chromatography with tandem mass spectrometry (LC-MS/MS) and liquid chromatography with fluorescence detection (LC-FLD). Other methods based on immunoassays are under development and variations of these and minor techniques are available for specific purposes.

## 1. Introduction

### 1.1. Ergot Alkaloids

The analysis of ergot alkaloids (EA) is of considerable importance, and a substantial topic because the alkaloids are encountered in many different situations that affect humans and animals, and they are found in many different matrices. The earliest interest was in the analysis of grain crops contaminated by the sclerotia (ergots) of *Claviceps* species and was based on the physical counting of the sclerotia in grain samples. Investigations led to the development of pharmaceutical forms of EA and subsequently the need for medicinal analysis, and in other cases, of forensic application. Developments in instrumental techniques have given us the ability to separate and measure individual ergot compounds and their isomers, and this in turn has allowed the possibility of monitoring and regulating the contamination of cereal based foods. There is a requirement therefore to measure EA in ergot sclerotia, infected cereals, forage grasses, processed foods, pharmaceutical preparations, illicit preparations, and body fluids and organs.

Chemical analysis today usually follows a distinct pathway of careful sampling and homogenisation, extraction of the analyte, separation of the analyte from co-extracted materials (clean-up), detection and quantification. Examples of these procedure are provided in the following paragraphs. Once a procedure has been developed its performance is characterised by repeated testing within a laboratory and then in ideal circumstances by a collaborative trial involving the participation of a suitable number of laboratories. Measurements can be qualitative (*i.e.*, identification of the EA present) or quantitative (*i.e.*, identification along with an estimation of the concentration present) depending on requirements and available reference standards. Several techniques are applicable to different matrices with some modification. There is a growing demand for rapid testing methods, and those that can be deployed in the field or on the production line.

There are three main classes of EA—short chain substituted amides of lysergic acid, clavine alkaloids (relatively insignificant substituted 6,8-dimethylergolines), and ergopeptines, which are peptide EA comprising (+)-lysergic acid and a tripeptide system containing L-proline. *Claviceps purpurea* produces lactam ergot alkaloids (ergopeptams) containing isoleucine as a second amino acid, which have been found to predominate in some infected wild grasses from Norway [1].

Methods for the determination of EA in cereals and their products were reviewed in 2001 by Komarova and Tolkachev 2001 [2] and again in 2008 by Krska and Crews [3]. Chromatographic and mass spectrometric methods to determine lysergic acid diethylamide (LSD) and related compounds in body fluids have been reviewed by Reuschel *et al.* [4].

**Figure 1:**
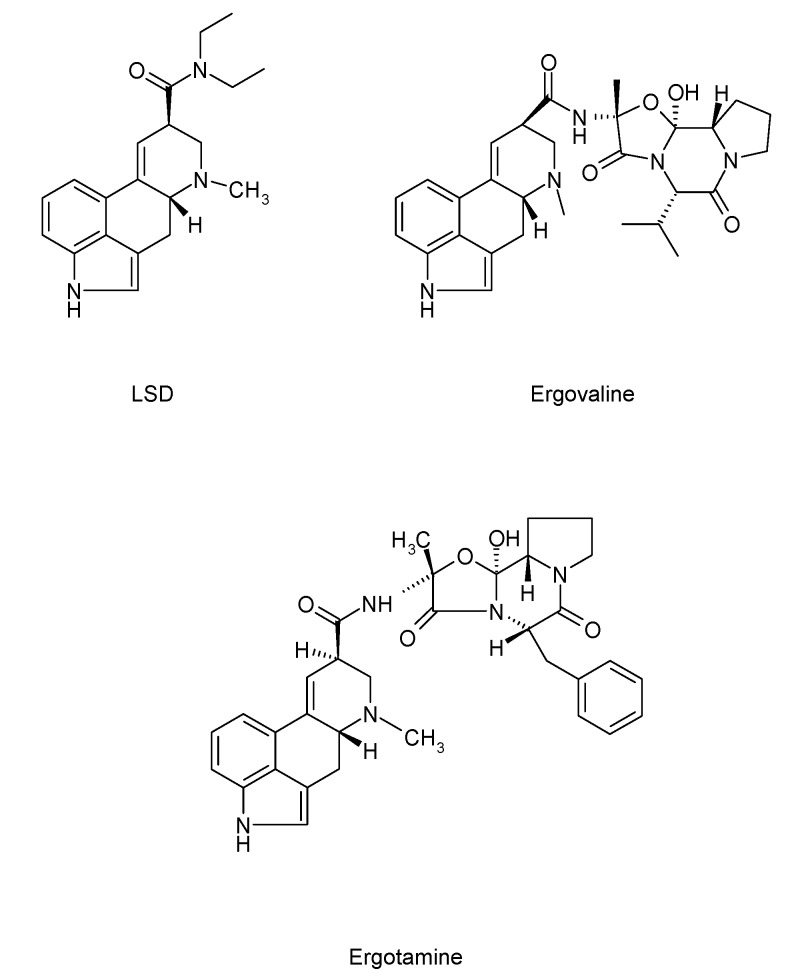
Structures of representative types of some major ergot alkaloids (EA).

Structures of representative types of some major EA are given in Figure 1.

### 1.2. Lysergic Acid Amides

Lysergic acid amides include ergonovine (ergometrine, or ergobasine), lysergic acid amide, lysergic acid diethylamide, lysergic acid 2-hydroxyethylamide, methylergonovine and methysergide. Most of these compounds are pharmacologically active. Lysergic acid is a chiral compound with two stereocenters. The isomer with inverted configuration at C-8 close to the carboxyl group is called *iso*-lysergic acid. Bromocriptine is a semi-synthetic ergopeptide used to treat Parkinson’s disease and a range of other illnesses.

### 1.3. Ergopeptines

**Figure 2:**
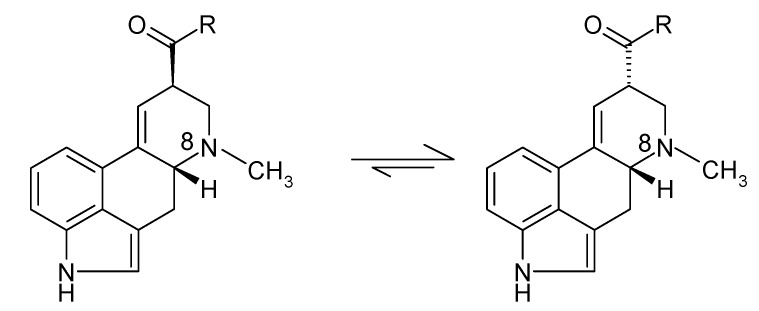
Epimerisation at the EA C8 position.

The EA found in *Claviceps* sclerotia are the ergopeptines ergometrine, ergotamine, ergosine, ergocristine, ergocryptine and ergocornine. These alkaloids have a double bond at C9–C10 which allows epimerisation to take place at the C8 position (as shown in Figure 2).

Epimerisation forms C8-(*R*) isomers with left-hand rotation, which are known as ergopeptines, and C8-(*S*) isomers with right-hand rotation, which are known as ergopeptinines, and are less toxic. The N6 nitrogen gives the protonated EA pKa values of 5.0 to 7.4, they have a positive charge in acid solution and are neutral in alkali. The driving forces for the conversion between epimeric forms are complex and not fully understood [5].

Ergovaline is a major ergot alkaloid found in tall fescue grass (*Fescue arundinacea* Schreb.) that has been infected with *Neotyphodium coenophialum*. Endophyte-infected tall fescue also contains ergovalinine, ergosine, ergonine, ergotamine, ergocristine, α-ergocryptine, β-ergocryptine, and ergocornine; the ergolines ergonovine, lysergol, and lysergic acid amide; and the clavines chanoclavine, agroclavine, penniclavine, elymoclavine, and 6,7-secoagroclavine [6]. Ergovaline and the associated EA in infected fescue cause sickness in livestock. The view has been expressed that ergovaline’s predominance in the fescue alkaloid pattern is due to the failure of some analytical methods to determine the simpler ergoline alkaloids present [7].

In the case of ergot alkaloid contamination of food and feed, the EU Scientific Panel on Contaminants in the Food Chain of the European Food Safety Authority (EFSA) has recommended that the major ergopeptines and their corresponding epimers (ergo-inines) are targeted for determination in food and feed to provide data to enable consumer exposure calculations [8]. Separation and determination of both epimeric forms is important as there are differences in their toxicity. The 12 priority alkaloids that EFSA recommends monitoring of are the main toxins in *Claviceps purpurea* (ergometrine, ergotamine, ergosine, ergocristine, ergocryptine, ergocornine), and their corresponding -inine epimers, and also dihydroergosine and agroclavine from *C. africana* and *C. fusiformis*.

EFSA has established a Tolerable Daily Intake (TDI) for EA of 0.6 μg/kg b.w. per day, a figure that can be closely approached by toddlers consuming a high proportion of cereals in their diet. Levels of EA in surveys of cereals and flour (particularly rye) have been reported at over 7000 µg/kg [3]. On this account accurate quantitative monitoring of EA in cereal foods and ingredients is clearly required. There are currently no legislated limits for total or individual ergot alkaloids in food, however it is likely that limits for EA will be included in future mycotoxin legislation. In Canada there are guideline limits for total ergot alkaloids in feed for poultry (100 µg/kg), feed for swine (6000 µg/kg), and feed for chicks (9000 µg/kg), and Uruguay has set a limit for ergot alkaloids in feeds of 450 µg/kg [9].

## 2. Stability

Natural samples always contain both EA epimers, and epimerisation is induced by various conditions of high or low pH, and strong light. The phenomenon has been studied in some detail in order to minimise changes in the natural ratio of epimers during storage, handling and analysis. The EA molecule has two keto forms (*R*- and *S*-epimer) which are interconvertible. Andrae *et al.* [5] proposed that in the conversion between the *R*- and *S*-epimer, the EA molecule passes through an intermediate conformation (enol-intermediate, see Figure 2). The authors used complex simulations to model the epimerisation process of ergocornine and α-ergocryptine and compared the models to the kinetic data of epimerisation obtained by experiment using two extraction solvent systems based on acetonitrile:water mixtures with ammonium carbamate. Quantum chemical models showed that the *S*-epimer is preferred in ergocornine, and in α-ergocryptine the distribution of its *R*- and *S*-epimers is balanced. Not all of the model behaviour was matched by experiment, showing that the epimerisation process is not yet well understood.

EA standards are best stored below −20 °C in non protic solvents or in the form of thin dry films [10], which has been shown to be suitable over a period in excess of 12 months [11]. Solutions of EA standards in ethanol containing tartaric acid have also been recommended [12] as have solutions in 25% ethanol containing ethylene glycol, 2-propanediol and tartaric acid [13].

In an alternative approach used in the determination of EA in cell extracts, analysis was preceded by incubating the EA in the cell culture medium until an equilibrium between the epimers was reached, a process that took up to 24 h at 37 °C [14]. Equilibration times differed with the alkaloid, with ergometrine being the most stable lysergic acid amide and ergosine the most stable peptide alkaloid. From the equilibration data correction factors were applied to quantify the biologically active -ine form.

LSD also undergoes epimerisation at the C-8 carbon forming the isomer *iso*-LSD [15]. The reaction is rapid in aqueous alkali and also occurs in alcohols and so LSD solutions should not be prepared in methanol or water [16]. It can be degraded rapidly on exposure to light of certain wavelengths, and in particular to ultraviolet irradiation, which promotes hydration of the C–9,10 double bond. Storage of samples such as urine or plasma in clear glass leads to a rapid loss of LSD but the use of amber glass or in polyethylene preserves the LSD for several days or weeks, especially at lower than ambient temperatures [16]. It has been reported to be stable for three months in urine kept at −20 °C [17].

## 3. Sampling

The first decision facing the analyst is that of the quantity of sample to be taken for analysis. In some medical and forensic applications this is often restricted by the quantity of matrix available, but for cereals and foodstuffs the sample size can usually be much greater. This is particularly important for EA on account of the very high concentration of alkaloids in some sclerotia. A cereal sample can contain a tiny fragment of sclerotia as a concentrated source of alkaloid within a bulk sample that is otherwise alkaloid-free. For large stores of grain a probe is used to take several portions from different locations, which can subsequently be blended and a representative subsample taken for analysis. Before extraction the samples must be reduced to a small particle size and thoroughly homogenised.

## 4. Extraction

EA can be extracted from dry samples (including sclerotia, cereals or grain-based foods) using relatively low polarity solvent mixtures with the addition of ammonium hydroxide to give an alkaline pH [18,19,20,21]. The solvents used typically contain ethyl acetate or dichloromethane (although the latter is out of favour on account of its toxicity and damaging environmental effects). An alternative approach is to use polar solvents such as methanol or acetonitrile mixed with dilute acid or buffer at a low pH [19,22,23].

Several comparisons of solvent systems have been made with complementary but slightly different results. Krska *et al.* [24] found that acetonitrile mixtures with ammonium carbonate buffer (84 + 16, *v*/*v*) extracted higher concentrations of EA than methanol acidified with phosphoric acid, or acetonitrile with ammonium acetate (1 + 2) at neutral pH. An acetonitrile-ammonium carbonate buffer (84 + 16, *v*/*v*) extraction solvent gave recoveries of about 90% to 120%. Adjusting the sample:solvent ratios from 1:3 to 1:10 (*w*/*v*) and varying the extraction times from 30 to 90 min did not significantly affect the recovery of the twelve EFSA priority EA from samples of rye and barley. For semi-quantitative and screening analysis less complex extraction solvent systems and procedures have been used, for examples Blaney *et al.* [25] simply homogenized sclerotia in methanol prior to high performance liquid chromatography with fluorescence detection (LC-FLD).

Spiering *et al.* [26] compared the effect of different solvents (acetic acid *vs*. chloroform-methanol ammonia, or 2-propanol-lactic acid) on isomerisation of EA during the extraction of ergovaline from tall fescue grass. Acetic acid caused isomerization of the ergotamine internal standard whereas this was more stable in the other solvents. For the 2-propanol-lactic acid solvent the optimum solvent was 2-propanol at 50% in water with the lactic acid concentration could be varied between 1% and 10% (*w*/*v*) with little adverse effect.

Extraction procedures based on extraction with organic solvent followed by partition between the solvent and a salt solution and then a dispersive solid phase extraction technique (QuEChERS) have become routinely used in contaminants analysis. A QuEChERS based method was first applied to ergot alkaloids by Malachova *et al.* [27]. Recoveries of ergocornine, ergocristine, ergocryptine, and ergosine ranged from 60% to 70%. The alkaloids were not detected in 116 cereal samples. A QuEChERS procedure was optimised for the extraction of ergovaline from tall fescue seed and straw for subsequent separation and determination by LC-FLD by vortexing the sample with ammonium carbonate/acetonitrile 1:1 *v*/*v* before adding magnesium sulphate and sodium chloride and vortexing again [28]. Following centrifugation an aliquot of the separated acetonitrile phase was evaporated to dryness and the extract reconstituted in methanol. Mean recoveries ranged from about 90% to 98%.

The effectiveness of four different extraction procedures for the simultaneous determination of 32 mycotoxins in barley, of which four were ergot alkaloids was compared by the analysis of a range of mycotoxins including some EA [29]. A blank barley sample was spiked with 100 µg/kg each of ergosine, ergocornine, ergocryptine and ergocristine. The methods compared were a modified QuEChERS procedure, matrix solid-phase dispersion (MSPD), solid-liquid extraction (SLE) and solid-phase extraction (SPE). The extracts were analysed by UHPLC-Orbitrap mass spectrometry.

For MSPD the homogenized barley was blended with either C8 or C18 phases for 5 min and the mixture packed into a glass column. The mycotoxins were eluted with 1 mM ammonium formate in 10 mL of acetonitrile/methanol (50/50, *v*/*v*). An aliquot of the extract was filtered for UPLC-Orbitrap MS. For the QuEChERS procedure the sample was steeped in 0.1% aqueous formic acid before the addition of acetonitrile and shaking. Then MgSO_4_ and NaCl were added and the mixture was shaken again before centrifugation and UPLC-MS. For SLE samples were extracted by shaking with acetonitrile/water/acetic acid (79:20:1 *v*/*v*/*v*) for 90 min, and then centrifuged, diluted with water, and filtered prior to UPLC-MS. The SPE clean up was applied to the SLE extracts, comparing C18 and HLB phases.

The QuEChERS method was preferred for multi-toxin analysis as it gave satisfactory recoveries, over 60% for most mycotoxins over a range of spiking levels (25 to 100 µg/kg) and was fast, inexpensive and could easily be modified and adapted. However for the EA included in the method the highest recoveries, about 80% to 91% were obtained with the SLE method used without SPE clean-up (a dilute-and-shoot approach). Recoveries with C8 and C18 SPE were lower than those without SPE, and the HLB phase was unsatisfactory for EA.

Analytical methods have been applied to animal tissues and fluids rather less frequently than those used for cereals, and procedures are generally less quantitative and less sophisticated. Liquid–liquid [30] or solid-liquid [31,32] extraction into diethyl ether or 1-chlorobutane are favoured approaches. Methods for the extraction of LSD from body fluids (urine, blood or plasma) is challenging because the ingested dose is normally very low (less than 250 µg) and the drug is rapidly metabolised. In urine samples the LSD concentration falls to <1 µg/L in a few hours. Where LSD can be detected in urine its metabolites such as *N*-desmethyl-LSD (*nor*-LSD) and 2-oxo-3-hydroxy-LSD are also present, the latter at higher concentrations than LSD. 13-Hydroxy-LSD and 14-hydroxy-LSD are excreted in urine, as glucuronide conjugates, but techniques (enzyme cleavage) to determine LSD in this form are not used routinely. The extraction procedures for LSD and its simple relatives typically involve a single liquid–liquid extraction of a small volume (e.g., 1 mL) of blood or urine (adjusted to alkaline pH) and direct analysis by gas chromatographic methods or transfer of the analytes into the LC mobile phase. Prior to extraction, blood can be clarified by addition of acetonitrile to precipitate material such as protein. Interestingly, conversion of *iso*-LSD to LSD by treatment with alkali has been used to increase the signal for the total LSD concentration for measurement by gas chromatography with mass spectrometry (GC-MS) [33].

## 5. Clean-up

Extracts of food, feed and biological tissues that will subsequently be analysed by chromatographic means are usually treated to separate the EA from other compounds extracted by the solvent. This serves several functions. It reduces the quantity of material placed on to the column which might otherwise affect the chromatography, it reduces the quantity of material reaching the detector which can dramatically affect sensitivity, and it can offer the potential for concentration of the analyte and for changing of the solvent composition. This ‘clean-up’ step is carried out using Solid Phase Extraction (SPE) in which small columns (cartridges) of material are used that temporarily and selectively binds the EA whilst most other compounds are flushed away with solvent. The EA are themselves then recovered by elution with a solvent of different composition which in turn leaves other co-extracted compounds bound to the column.

SPE clean-up based on various different chemistries can be applied to the clean-up of EA extracts, including basic alumina cartridges, C18 reversed phase, Hydrophilic-Lipophilic Balance (HLB), strong cation exchange (SCX), mixed-mode cation exchange (MCX) cartridges and immunoaffinity methods using immunoaffinity columns (IAC). In a typical alumina SPE clean up, Müller *et al.* [18] extracted EA from flour and rye by shaking with a mixture of ethyl acetate, methanol and aqueous ammonia. An aliquot of the supernatant was passed through a basic alumina column and the eluate collected for transfer into LC solvent. For C18 clean up Mohamed *et al.* [34] passed an extract in acetonitrile/ammonium acetate through a cartridge and eluted EA with methanol/acetonitrile. For SCX Storm *et al.* [22] applied EA in an extracting solution comprising methanol and aqueous phosphoric acid, and after washing the phase eluted the alkaloids with a mixture of methanol and ammonium acetate at pH 10.2 prior to separation and detection by LC-FLD.

An improved SPE method [35] used SCX material neutralised with sodium (Na^+^-SCX). This enabled a neutral extraction solvent to be used. EA (in their protonated form) were eluted from the column by forming ion pairs with sodium hexanesulfonate, which delayed epimerization for over 96 h. The method has been tested with the 12 EFSA priority EA and also applied to rye flour and an acetone extract of wheat germ oil. Detection by LC-FLD showed the method to perform well for both the oil and rye flour.

In a comparison of the performance of reversed-phase SPE cartridges intended for basic compounds, mixed-mode MCX, and HLB sorbents, good recoveries were obtained with negligible epimerisation with the HLB column for all EA except ergometrine which was not sufficiently retained on the column due to its higher polarity [36]. With MCX cartridges all of 12 EA tested were recovered quantitatively despite not being added in an acid phase, a normal requirement for an ion-exchange mechanism. Retention of the EA on this column phase was ascribed to a degree of reversed-phase activity in the column phase and/or the presence of a charge on molecules even under the neutral or slightly alkaline conditions. Elution from the MCX cartridge with 5% ammonia as normally applied induced considerable epimerisation to the -inines. Others have reported relatively poor performance of SPE materials of various types including aminopropyl, HLB and SCX phases [34,37].

A dispersive PSA-SPE procedure was applied by Krska *et al.* [36] in which polar matrix compounds are bound to an ethylenediamine-based primary and secondary amine (PSA) added directly to the acetonitrile/ammonium carbonate buffer extract. The procedure is similar to SPE methodology, but the sorbent is not held in a cartridge but added directly to the extract, mixed and then removed by filtration. The PSA phase is a weak anion exchanger that absorbs many hydrogen bond forming co-extractives from the matrix. As SPE conditioning and washing are not required the method is very rapid.

Molecularly imprinted polymers have been developed for use in SPE prior to tandem mass spectrometry (LC-MS/MS) [38]. The imprinted polymer, produced on suspended polymerized beads with a metergoline template, could recognise and trap six EA and their epimers from solution. Recently, silica has been chemically functionalised with aminated DNA as an aptamer DNA ligand specific for ergot alkaloid [39]. Extracts of rye feed extract were added to aptamer functionalised silica gel, mixed for one hour and then centrifuged. The supernatant was discarded and the silica gel washed with water. EA were eluted from the silica with dilute hydrochloric acid, and separated by centrifugation for LC-MS/MS. In a limited application the aptamers were very specific for certain EA (ergosine, ergocryptine, and ergocornine) but perhaps did not trap ergotamine, ergometrine or ergocristine if these were present. It was concluded that aptamers are more specific than MIPs and can be used to extract targeted EA, with the advantage of being robust and (unlike antibodies for IACs) not requiring the use of animals in their production.

Modern LC-MS/MS instruments have such sensitivity that instead of using sometimes complicated techniques to clean up the extract it can be injected directly after considerable dilution. This simple action can reduce the matrix effect and so, paradoxically, improve sensitivity despite the dilution effect. Dilution is usually made using the LC-MS mobile phase [23,40]. This simple approach has been used to recover EA from beer as part of a multi-mycotoxin method in which undesirable co-extracted compounds were precipitated by adding acetonitrile prior to direct injection [41].

## 6. Analytical Methods

### 6.1. Capillary Electrophoresis

Capillary zone electrophoresis (CZE) has been applied to determine EA and some epimers using β- and γ-cyclodextrins, urea and poly(vinyl alcohol) in phosphate buffer at pH 2.5 with a fused-silica capillary at 25 kV [42]. Good separation was achieved, but for a limited number of alkaloids. UV and fluorescence detection methods were used. CZE has been used to determine lysergic acid, *iso*-lysergic acid and the related paspalic acid in reaction mixtures [43]. The method was made compatible with mass spectrometric Time of Flight (TOF) detection as well as UV by optimising the running background electrolytes (BGEs) which comprised methanol with asparagine, sodium tetraborate, or ammonium acetate at alkaline pH. The LODs were below 0.5 mg·L^−1^ with UV detection and below 0.1 mg·L^−1^ with TOF. These LoDs are inferior to those of LC-MS/MS-based methods.

### 6.2. Gas Chromatography-Mass Spectrometry

Ergopeptides are non-volatile and susceptible to heat, therefore gas-chromatographic methods cannot be used on the EA directly. GC-MS can however be applied to the analysis of lysergic acid amides and low molecular weight clavines following chemical reaction (derivatisation) to improve volatility and stability by blocking polar groups. Trifluoroacetyl (TFA) derivatives of LSD and *iso*-LSD are prepared by heating the dry sample with trifluoroacetic anhydride or with trifluoroacetylimidazole at 70–80 °C for about 30 min. *N-*Trimethylsilyl (TMS) derivatives are made in similar fashion by heating the dry sample with *N*,*O*-bis(trimethylsilyl)trifluoroacetamide (BSTFA) containing 1% trimethylchlorosilane (TMCS), or with methyltrimethylsilyltrifluoroacetamide (MSTFA). The reaction mixtures from each of these derivatisation procedures can be injected directly into the GC. The TFA derivatives of *iso*-LSD and LSD coelute to some extent, making identification and quantification difficult when both are present. The TMS derivatives are however baseline resolved. For examples of the determination of LSD by GC-MS the reader is referred to the relevant publications [44,45,46,47].

GC-MS methods include electron impact (EI) ionisation in which a number of fragment ions are produced from the ionised analyte, and tandem (GC-MS/MS) methods in which chemical ionisation (CI) in positive or negative mode is used to give an intense molecular ion which is subsequently fragmented by collision to give fewer specific ions of high intensity. GC-MS in EI mode of the TMS derivatives of LSD and *nor-*LSD produces ions suitable for selected ion monitoring (SIM) regimes at the following *m*/*z* values: LSD-TMS: *m*/*z* 395, 293, 268 and 253; *nor-*LSD-TMS: *m*/*z* 381, 279 and 254 [48]. However the dissociations induced by GC-MS/MS techniques provide much greater sensitivity combined with the opportunity to confirm peak identities. The pathways of fragmentation in GC-MS/MS have been reported in some detail [44,48]. Chemical ionization is used in MS/MS experiments as it produces more intense precursor ions than electron ionization as fragmentation is much reduced.

The fragmentation of the negative-ion ammonia and methane CI precursor ions of LSD and *N*-demethyl-LSD as TFA derivatives and also fragmentations of the positive-ion CI of their TMS derivatives have been compared [48]. The TFA derivatives of LSD, *iso*-LSD and the reference standard lysergic acid methylpropylamide (LAMPA) primarily lost a fragment of 97 mu, corresponding to loss of trifluoroacetyl from the N-1 position with only small differences in the relative product ion abundances. The signal at *m*/*z* 404 (loss of trifluoroacetyl) was 10-20-fold higher for the TFA derivative of *N*-demethyl-LSD than the corresponding signal for LSD (*m*/*z* 322), allowing the *N*-demethyl-metabolite to be quantified at lower levels, typically down to 50 pg/mL in urine. For the TMS derivatives however the parent compound could be detected at lower levels than *N*-demethyl-LSD, possibly because of inefficient derivatisation of the latter. The principal eliminations from the dissociation of protonated LSD and LSD-TMS involve losses of CH_3_ radical, CH_3_NH_2_, CH_2_NCH_3_, diethylamine, diethylformamide ((CH_3_–CH_2_)_2_NCHO), and *N*-diethylpropenamide from the protonate molecular ion.

### 6.3. Liquid Chromatography

Reverse phase-based chromatography is always used for the separation of EA. Most methods use solvent systems of methanol-water or acetonitrile-water mixtures with added ammonium hydroxide, ammonium carbonate, ammonium carbamate or triethylamine to provide alkaline pH conditions. Separation can be achieved with both isocratic and gradient mobile phases. Alkaline mobile phases are preferred to maintain the stability of both epimers, to avoid protonation and to improve separation. The six major *C. purpurea* alkaloids and their epimers can readily be baseline separated by high performance LC within a short run time, with individual ergopeptines eluting immediately before the corresponding ergopeptinines in alkaline mobile phases. The α- and β- epimers of ergocryptinine, and the pair β-ergocryptine and ergocristine are not always separated, although with LC-MS/MS detection ergocryptine and ergocristine can be distinguished by their different masses. It is important to inject the sample in solution in a solvent of similar composition to the starting mobile phase as sample solvents that are more polar than the mobile phase can cause distortion of the chromatographic peaks [37]. By the use of ultra-high performance liquid chromatography (UPLC) the chromatographic run time can be as short as 5 min [19].

### 6.4. Ultraviolet and Fluorescence Detection

With LC separation ergopeptines and ergopeptinines can both be measured with an ultraviolet (LC-UV) detector set to a wavelength maxima of 310 nm for ergopeptines and ergopeptinines and at 280 nm for dihydroergopeptines, although other wavelengths have been included [25].

Many EA, including the most common, are naturally fluorescent [2], and as LC-FLD offers a higher sensitivity for many EA compared to LC-UV, its use is much in favour [11,14,18,20,22,49,50]. The best excitation and detection wavelengths are 310 nm and 410 nm respectively for Δ9,10-ergolenes, and 272 nm and 371 nm respectively for Δ8,9-EA and EA with a saturated D-ring, although other wavelengths have been used. LC-FLD can be used to determine all 12 of the EFSA-named epimeric EA providing sufficient chromatographic resolution is provided. Where this is not demonstrated some compounds, especially α- and β-ergocryptine and similarly α- and β-ergocryptinine might be reported as single compounds if they coelute.

### 6.5. Mass Spectrometry

#### 6.5.1. Introduction

Determination by high performance liquid chromatography (HPLC) coupled with tandem mass spectrometry (LC-MS/MS) has become a standard approach for trace quantification and identification. In this technique alkaloids individually separated by HPLC are ionised in an electrospray (ESI) interface to produce a protonated molecular ion that is made to undergo collision with gas molecules to induce fragmentation into further charged “product ions” that can be separated and identified in the final detection stage. EA are more easily ionised in electrospray interfaces of mass spectrometric sources than in the alternative atmospheric pressure chemical ionisation (APCI) on account of their polar nature. Operation in ESI-positive mode (ESI+) usually gives more intense ions (protonated molecular ions [M+H]^+^) than the deprotonated molecular ions ([M−H]^−^) obtained in the ESI-negative mode (ESI^-^) [34]. As well as protonation (formally, proton adducts) MS ionisation is often accompanied by the formation of adducts with other cations such as sodium [M+Na]^+^ present in the aqueous mobile phase and/or the ammonium [M+NH_4_]^+^ component of buffers. However in EA ionisation these adducts have low intensity compared to the protonated molecular ion.

Sophisticated methods are used to identify fragments and unknown whole alkaloids. The major ions produced by the fragmentation of the important EA has been fairly well identified using stepwise fragmentation experiments (MS*^n^*) experiments in LC-MS/MS, and their structures have been elucidated. Detailed descriptions of the known and postulated fragmentation routes have been provided [51,52,53,54]. The ESI mass spectra of the peptide EA have protonated molecular ions and fragment initially through loss of water (M+H−18)^+^ from the C-12' alpha-hydroxy group and then through loss of other fragments. In ergovaline and ergotamine the amide and ether groups are cleaved from ring E but the peptide ring’s methyl group is retained, giving a fragment at *m*/*z* 320. An equivalent fragmentation of ergocornine, ergocryptine and ergocrystine, with the retention of the *iso-*propyl group forms a fragment with *m*/*z* 348 [51]. Characteristic ions including common product ions at *m*/*z* 208, 223, 225, 251 and 268 arise from successive losses of NH_3_, CO, and CH_3_. Dihydroergotamine has equivalent fragment ions at *m*/*z* 253 and 210. The major ions produced by ergopeptines are identical to those of their ergopeptinine epimers. Ergopeptams do not undergo the loss of water but produce an intense fragment at *m*/*z* 350 and weaker ions at *m*/*z* 322 and *m*/*z* 251 in succession [53].

A common abundant product ion such as that with *m*/*z* 223 can be selected and its parent molecule identified, or the ion subjected to further collision induced dissociation. Applying these techniques to partially deuterated alkaloids can help to identify the location of dissociation sites [52].

In quantitative analysis the selection and monitoring of relatively intense and specific fragment ions in LC-MS/MS is used to both confirm the identity of the EA, and confirm the quantification. The protonated or deprotonated molecular ion is used for the primary quantification process as it is usually the most intense. The ratios of this to the selected fragment ions (transition ions) provide confirmation when compared to the ratio for standards. If this ratio does not agree, within a certain tolerance, with the ratio obtained for an authentic standard, then this indicates either: that the putative peak is not that EA, or that the LC peak also contains other interfering substances; and therefore the quantitation is suspect. Specific transitions typically used include [M+H]^+^ → *m*/*z* = 268, 223 and/or 208 [24,52]. Transitions of the protonated precursor ion (M+H^+^) to *m*/*z* 223 are frequently used as the quantifier ion with the transitions to *m*/*z* 208 as a qualifier. For ergocryptine and ergocristine the transition of M+H^+^ to *m*/*z* 268 as qualifier is more appropriate.

To increase sensitivity the acquisition of data for the individual alkaloids is usually divided into two or more time windows in each of which only transitions of the EA eluted in that window are recorded. This allows for more rapid and frequent sampling by the mass analyser leading to both an increase in signal size and a better measurement of narrow peaks. This has become more important with the availability of very high performance LC columns that can separate EA within a short period reducing the elution duration of each.

Time of Flight mass spectrometry is a detection method for LC (LC-TOF-MS) that gives very high mass resolution (>20,000) and thus provides a low level of background noise and the associated high sensitivity as better signal:noise ratio. A further advantage of the method is that the acquisition of data is non-targeted (the whole mass range is monitored and recorded unlike the few selected masses monitored in LC-MS/MS) and so spectral information for a large number of compounds can be extracted from a single LC run. Since all of the mass data are recorded then the technique has the additional advantage that the files can be inspected retrospectively in discovery mode, to look for novel EA, metabolites or any other substance that was not even thought about at the outset. The method is somewhat less sensitive than MS/MS methods and has thus seen little application in EA determination to date. However, using a quadrupole-time-of-flight mass spectrometer (LC-QTOF-MS) multiple MS/MS experiments (MS*^n^*) can be carried out to provide more specificity, sensitivity and detailed results. Rouah-Martin *et al.* [39] demonstrated the application of LC-QTOF-MS in positive ion mode to determine EA in a small number of rye extracts cleaned up using an immobilised aptamer. Theoretical monoisotopic masses were calculated and compared with the experimental data. Two samples contained ergosine at (*m*/*z* 547.2874) and ergocryptine (*m*/*z* 575.3085), and a second sample also contained ergocornine (*m*/*z* 561.2916). All alkaloid peak spectra contained the characteristic precursor fragment at *m*/*z* 223.1283.

#### 6.5.2. Matrix Effects

Mass spectrometric detection following LC in particular is prone to alterations of the signal intensity caused by the presence of co-extracted compounds in the instrument—so called matrix effects. Matrix derived compounds can affect the ionisation efficiency in LC-MS, leading to signal enhancement, or more usually suppression. These problems can be addressed to some extent by using calibration standards prepared in a matrix extracted from a “blank” sample (matrix-matched calibration) but optimisation of the extraction and clean-up has an important role.

Matrix effects can be compensated for to a large extent by the use of isotopically labelled internal standards but care must be taken as interferences can easily influence the response for the *m*/*z* value of an analyte but not the slightly different *m*/*z* value of its isotopically labelled analogue.

A systematic study has been made of the matrix effects in the LC-MS/MS analysis of EA in cereals with the aim of eliminating or minimising their effect for a range of cereals [55]. The recovery of EA was not significantly affected by the extraction conditions, but clean-up and detection methods were important factors. Signal intensity was suppressed by the matrix more strongly for the early eluting compounds, with a significant difference between the grain types. With ergometrine in barley and oats suppression reached up to 90% and for ergocryptinine and ergocristinine in oats and barley suppression was up to 50%. Three varieties of rye each exhibited different matrix effects. Therefore the blank matrix material used for the preparation of matrix matched standards must be carefully chosen. The bestmatrix-match approach is the method of standard additions where the sample extract itself provides the matrix for the calibration standards (see later). Ergometrine and ergometrinine recoveries were most affected by sample preparation procedures. MycoSep^®^ multifunctional SPE reduced matrix more significantly than other media but recovery of ergometrine was still poor. For most EA very high enhancement of the signal intensity was observed under APCI when compared to ESI.

## 7. Identification and Quantification

Criteria for the analysis and quantification of contaminants are described in regulations, the most relevant for the determination of EA in food and feed in Europe being those set out in the Directive 2002/657/EC [56]. Positive identification of EA can be assured when certain conditions have been met. These include having the LC retention time for a sample EA peak agree within a narrow range with that of the standard, and a response for both the quantifier and qualifier ions, the peak areas of which are in a ratio that agrees with those of the standard.

The usual quantification procedure is to compare the response for the samples against a calibration series prepared in blank matrix to compensate for matrix effects. As the matrix effect for the samples may differ from that for the standards a more accurate measurement can be made by a multi-point standard additions technique where a number of standards (usually five or more) are spiked into the sample and the quantity present in the unspiked sample is calculated from the intercept. This has a major drawback of requiring many separate analyses of the same sample. Mülder *et al.* [23] modified this approach to quantify EA in cereal samples by screening by means of a duplicate analysis in which one subsample was spiked with EA to enable an approximate calculation of the concentration to be made. This is effectively a single-point standard addition. Where this showed samples to contain a significant quantity of alkaloids they were then determined accurately by means of multi-level standard additions.

Internal standards are ideally used for quantification in LC-MS and LC-FLD procedures but to date no isotopically labelled standards are commercially available for the LC-MS analysis of most of the priority alkaloids named by EFSA. In the case of LSD deuterated analogues (d3-, d6- and d10-LSD) are available with the former most commonly used in forensic applications [46,57,58]. The analogues lysergic acid methylpropylamide [59], and *N*,*N*-methylpropylamide [60] have been used. Care must be taken when using these deuterated LSD molecules as on MS fragmentation the deuterium atoms are absent in all of the significant ions other than the molecular ion, being lost with the cleaved diethylamide group. Since the (protonated) molecular ion is normally the most intense ion and is used for quantification (see above) then this is not a serious shortcoming. Methysergide maleate was used as an internal standard in the analysis of six major EA in human cells [14].

In the determination of 16 alkaloids in over 100 samples of bread, flour, infant formula and baby food the reliability of LC-MS/MS data was much improved by incorporation of dihydroergocristine as an internal standard [50]. Dihydroergocristine has also used been as an internal standard in the analysis of serum for dihydroergotamine [61].

Good quality primary standards are available commercially for the EFSA listed ergometrine, ergotamine, ergosine, ergocristine, ergocryptine, ergocornine, and their epimers, also for dihydroergosine and agroclavine, and also for the pharmaceutical compounds methysergide, dihydroergocristine, dihydroergosine, dihydroergotamine, bromocriptine, D-lysergic acid, various lysergic acid amides and ethylergometrine. Many countries impose licensing restrictions on the purchase and use of LSD and of EA that are precursors to its synthesis.

## 8. Multiresidue Mycotoxin Screening Methods

Ergot alkaloids are increasingly being incorporated into multimycotoxin methods. These techniques are very useful for screening cereal products but the extraction, chromatographic and ionisation conditions needed to increase the range of analytes may mean that results for the less robust toxins such as EA might be less reliable in quantitative terms. In many of the earlier multimycotoxin methods the inclusion of EA was restricted to a few particular compounds.

Multi-mycotoxin methods are an attractive option for the screening of contamination in large numbers of samples and there are advantages in including in the analyte suite as many of the commonly encountered mycotoxins as possible. This suite should certainly aim at including as many of the major ergot alkaloids as possible. However the inclusion of very many analytes of different structure into one method means that compound-specific clean-up step cannot be used without poor recovery of some compounds. To perform multianalyte determinations the sample preparation procedure must be as basic and simple as much as possible. For this to work effectively the mass spectrometric technique must have very high sensitivity and a low response to background signals.

These factors can be achieved with high resolution time-of flight (TOF) MS where the mass resolving power can distinguish between and analyte signal and one from a background compound of almost identical mass. Mass resolution has been much improved by the introduction of “Orbitrap” technology, which is a modified ion trap with a high resolving power and mass accuracy. The method has been applied to multimycotoxin analyses including ergot alkaloids [53] for such matrices as beer (for 32 mycotoxins) including seven ergot alkaloids [41].

The data from high resolution TOF acquisition with minimal clean-up covers the a wide spectral range including the ions of *m/z* below 100 up to *m/z* values well in excess of those of the ergot alkaloids. This offers a great advantage in that the data can be investigated (mined) at a later date to discover and measure compounds in addition to the target analytes

There are several recent examples of the inclusion of EA into multi-mycotoxin methods [62,63,64,65]. The advantage of multi-toxin methods is that samples can be tested for a wider range of potential contaminants with consequent economies of time and cost.

## 9. Immunological Methods

Immunoassay methods including Enzyme Linked Immunosorbent Assays (ELISA) have the advantages of being rapid and inexpensive but are less specific and less accurate than LC-FLD or LC-MS methods. ELISAs have been developed for EA and some are available commercially. ELISA is a rapid technique where large numbers of samples need analysis and is also relatively inexpensive as parallel analysis of several samples is routine, and expensive equipment is not required. ELISA methods are not specific for individual EA. This has the disadvantage that it is not possible to estimate the degree of toxicity from the results and the advantage that it might determine EA that are not targeted by chromatographic methods. Antibodies are raised that specifically bind to the lysergic acid ring structure, however many peptide alkaloids (ergocryptine, ergocristine, ergocornine, and ergotamine) have large groups attached to the lysergic acid which hinder the antibody binding and are therefore not amenable to ELISA. ELISAs have been used to determine ergovaline in seed and straw, and also in rumen fluid [66] but the concentration measured differed greatly and inconsistently when compared to an HPLC procedure. Excessively high results for contaminated fescue grass analysed by ELISA were attributed to the presence of setoclavine, an alkaloid produced by the oxidation of agroclavine [67]. With an expected increase in demand for rapid testing in the field the development and validation of dipstick type tests is urgently required.

Immunoassay methods are widely used to measure LSD in body fluids but are prone to giving false positive results or substantially higher quantification of LSD, presumably on account of cross-reactivity to other compounds [68].

Immunoaffinity based extraction and clean-up methods have been applied to LSD to produce samples suitable for determination by GC-MS or LC-MS and some extraction devices are now available commercially [68,69,70].

ELISAs are frequently applied to determine the total ergot alkaloid content of fescue grass forage [71,72]. Commercial test kits were used for this purpose [73] and also to determine EA in urine from lambs that had been fed with tall fescue [67].

Aptamers are artificial ligands that bind to target molecules with high specificity. Aptamers specific to EA have been produced by selecting DNA ligands with an iterative selection procedure. They are cloned and sequenced and the aptamer having the optimised dissociation constant is then linked to gold nanoparticles. A colorimetric reaction takes place in the presence of the target analyte. Aptamers were produced from the readily available metergoline and lysergamine as these had the common ergoline skeleton and were structurally similar to lysergic acid [74]. The selected aptamer was tested with lysergamine, metergoline, ergocornine and an extract of ergot contaminated flour that contained six major EA and their epimers. The aptamer allowed the detection of the smaller alkaloid molecules (lysergamine and ergometrine) without false positives from similar indole compounds present in foods (tryptophan, gramine and tryptamine).

## 10. Other Spectroscopic Methods

Various spectroscopic techniques have been applied to the detection and quantification of EA. The compounds form an intensely coloured blue solution on reaction with p-dimethylaminobenzaldehyde under acid conditions and illumination which can be measured at 580 nm. A modified version of this test is used to test the purity of salts of ergometrine and ergotamine [75]. Ninhydrin also reacts with EA [76]. Clearly these methods can provide only a value for the total EA content (sum parameter). Unfortunately several other natural alkaloids and amines (such as tryptophan) may also produce the colour reaction [77].

Ergot sclerotia can be detected and quantified in cereals using NIR hyperspectral imaging and multivariate image analysis [78]. Discrimination is based on the fat and starch content ergot bodies, which differ from those of cereals. The method is intended for use in cereal conveyor belt systems at an industrial level and has identified a sclerotia content of 0.02%, which is below the European Commission limits of 0.1% for feedstuffs containing unground cereals, and 0.05% in “intervention” cereals destined for human consumption. In addition, no false positives were obtained. The method avoided the common difficulty in visual methods of distinguishing sclerotia from similarly shaped and coloured objects and is far more rapid than the microscopy-based reference procedure.

## 11. Other Methods

The presence of ergot sclerotia can be detected qualitatively by determination of the presence of ricinoleic acid ((*R*)-12-hydroxy-(*Z*)-9-octadecenoic acid) which is a major fatty acid of ergot. The oil is extracted and the fatty acids measured by hydrolysis and derivatisation of the carboxyl group by silylation prior to GC with flame ionisation detection [20]. The ricinoleic acid content is however independent of the EA content.

## 12. Method Performance

Analytical methods are generally characterised by measurement of their performance in terms of the repeatability, the precision, accuracy, linearity, robustness, specificity, detection limit and Limit of Quantification (LOQ), which is the smallest amount of analyte in a test sample that can be quantitatively determined with suitable precision and accuracy under previously established method conditions [79]. The LOQ is of particular importance in setting the lowest level at which positive results are reported.

The reported performances of some recent methods applied to food and feed are provided in Table 1. Methods for LSD and its metabolites in blood and urine, which are more frequently qualitative, typically have limits of quantification lower than 20 pg/mL with good recovery and repeatability [80].

**Table 1:** Method performances of some recent methods applied to food and feed.

Matrix	Extraction solvent	SPE	Detection	LOD µg/kg	LOQ µg/kg	Recovery (%)	Repeatability precision (RSD%)	Reference
Cereals	MeCN acetic acid	PSA	MS/MS	NA	0.17–2.78	70–105	NA	[36]
Food and feed	EtOAc, MeOH, ammonium bicarbonate	phase separation	MS/MS	NA	0.1–1.0	50–90	mu 50%–30%	[37]
Rye	MeCN ammonium acetate pH 6.5	C18	MS/MS	7–11	23–37	24–92	7–12	[34]
Rye	MeCN, water	Na^+^SCX	FLD	NA	2.0	80–120	NA	[35]
Rye	EtOAc, MeOH, NH_3_	filter	FLD	NA	Max 3.3 max	89–100	3–15	[18]
Rye & wheat	MeCN ammonium carbonate	IAC	MS/MS	NA	0.01 to 0.06	80–120	1–14	[19]
Rye	Phosphoric acid	SCX	FLD	0.2–1.1	NA	50–70	NA	[22]

NA = Not Available.

## 13. Method Validation

Method validation can be carried out within laboratory or preferable by collaborative trial that is carefully designed to include a suitable number of proficient laboratories and includes analysis of appropriate samples for the desired analytes. Evaluation requires the correct handing of samples and standards. Elaboration of the results is enhanced by the availability of standard reference materials. Only a single certified reference material (rye matrix) is available for EA analysis [8].

An LC-FLD method to determine 12 EA in grain and flour was validated in an international trial with 26 participant laboratories in 2012 [12].

An official §64 method of the German Food and Feed Law (LFGB) which was also tested in an international trial with 26 laboratories has recently been validated and published for grain and flour [12]. This method is based on the method of Müller *et al.* [18] and uses ethyl acetate/methanol/ammonium hydroxide 25% (75/5/7; *v*/*v*/*v*) for extraction of a 20 g sample, basic alumina cartridges for sample clean-up and HPLC separation on phenyl-hexyl columns and FLD detection. The LOQ for this method ranged from 0.08 to 3.3 μg/kg with the highest (worst) LOQ for ergometrine.

Regular proficiency testing has indicated that results for the analysis of naturally contaminated rye have a relatively high standard deviation.

## 14. Specific Applications

### 14.1. Cereals, Food and Plant Tissues

LC-MS/MS has been widely applied to the analysis of cereals and processed cereal products such as rye bread. In a current procedure having good performance, EA were extracted from cereal and cereal samples by shaking for 30 min with a mixture of ethyl acetate/methanol/0.2 M pH 8.5 ammonium bicarbonate (62.5/25/12.5 by volume). The extract was centrifuged and solutions of ammonium bicarbonate and ammonium sulphate added to separate the ethyl acetate phase. The ethyl acetate layer was removed and the solvent evaporated, and the residue reconstituted in a mixture of methanol/acetonitrile/water (20/40/40 by volume). The reconstituted sample was washed with *n-*hexane before analysis by LC–MS/MS. For corn and grass silage samples the evaporated ethyl acetate layer was mixed with dichloromethane before addition of the ammonium bicarbonate and ammonium sulphate solutions. The analysis used LC–MS/MS. The C18 column was operated with a binary mixture mobile phase of A (water/methanol 85:10 *v*/*v*) and B (water/methanol (5:90 *v*/*v*) both with 5% 0.2 M ammonium bicarbonate at pH 10 operated with a gradient of 30% A decreasing to 0% A. The LC–MS/MS was operated in positive electrospray ionisation (ESI+) mode. Data were acquired in five different time windows in SRM mode with two transitions monitored for each analyte. The validated method had LOQs ranging from 0.1 to 1 µg/kg according to analyte and matrix. Recoveries were typically around 90% except for ergometrine in a biscuit matrix [37].

Rye flour was determined by LC-FLD after extraction under neutral conditions by shaking for 1 h with acetonitrile/water (84:16 *v*/*v*) [35]. Cleanup on sodium-neutralised strong cation exchange resin (Na^+^-SCX) was used to determine the 12 priority ergot alkaloids. EA were eluted from the (Na^+^-SCX) column as ion pairs with sodium hexanesulfonate. The method had reduced tendency to form epimers of the alkaloids but required in-house preparation of the resin. The LC-FLD determination was carried out using a phenyl-hexyl column operated under isocratic conditions with a 50:50 (*v*/*v*) mixture of water containing ammonium carbonate and acetonitrile. The detector was set at λex = 330 nm and λem = 415 nm. The method had recoveries of 80%−120% for rye flour and LOQs of 0.7−2.0 μg/kg per individual EA. It was also applied to edible oil matrices after extraction with acetone.

An SPE clean-up column for EA that worked on a convenient push-through principle enabled clean-up to be completed within one minute [19]. EA were extracted from rye and wheat grain by shaking for 1 h with acetonitrile/ammonium carbonate buffer (84:16, pH 8.5). The filtered extract was passed through a MycoSep^®^ 150 Ergot SPE clean-up column. The eluate was evaporated and the residue redissolved in the LCMS mobile phase. Using a UPLC C18 column with 1.7 µm particle size and a gradient of acetonitrile and ammonium carbonate buffer the total run time was only six minutes. Ten EA were determined by LC-MS/MS with LOQs ranging from 0.01 to 1.0 µg/kg (wheat) and 0.01 to 10.0 µg/kg (rye). Recoveries were mostly between 80% and 120% with good within-day repeatability (RSD < 14%).

EA were extracted from ergot sclerotia, cereals and feed by tumbling the powdered sample with a mixture of methanol and water with formic acid (600/400/4). The extract was simply centrifuged and filtered before LC-MS/MS in positive electrospray mode using the standard procedure of monitoring two transitions per compound. The method had an LOQ of 10 µg/kg for individual EA in cereals and 5 µg kg in sclerotia [23].

Ergovaline in endophyte infected grasses such as tall fescue is commonly determined by application of LC-FLD, the procedure developed by Rottinghaus *et al.* [81] often being applied [82]. Milled grass is extracted with chloroform made basic with sodium hydroxide, filtered with a phase separating paper and cleaned up with an Ergosil^®^ silica gel SPE column with the ergovaline eluted with methanol. Ergovaline is detected using an excitation wavelength of 250 nm and an emission wavelength of 420 nm.

In a typical acid extraction procedure for fluorescence detection [22] rye flour was extracted with methanol:0.013 M aq. phosphoric acid (70:30 *v*/*v*) by shaking for 30 min. The extracts were centrifuged and filtered through paper, and then mixed with 0.013 M aqueous phosphoric acid and filtered through a 0.2 µm filter. The extracts were cleaned up using conditioned SCX-SPE columns with EA being eluted with methanol:0.01 M ammonium acetate at pH 10.2. The solvent was evaporated and the residue dissolved in LC solvent. LC-FLD used a C18 column and a mobile phase of acetonitrile:aqueous 0.01 M ammonium carbamate 1:4 *v*/*v*, pH 9.6 (A) and acetonitrile (B). The fluorescence detector was operated with an excitation wavelength of 240 nm and an emission wavelength of 410 nm. The mobile phase pH was set to minimise interference from coeluted matrix peaks. Some significant C8-isomerization was observed at a compound-dependent rate in both sample extracts and standards.

Ergovaline was extracted from goat's milk with chloroform after precipitation of the milk proteins with acetone [83]. It was then cleaned up using an Ergosil^®^ column, a chemically modified silica gel designed to retain ergopeptine alkaloids. After washing the column phase with acetone–chloroform the ergovaline was eluted with methanol. LC separation was carried out with a C18 column with isocratic elution with acetonitrile–ammonium carbonate. Both fluorescence and MS/MS were used for detection and confirmation. Recovery of ergovaline from spiked milk was close to 100%.

### 14.2. Other EA

Other applications have included the analysis of fungal and plant extracts. EA were isolated from *Aspergillus* by LC-FLD prior to manual collection of individual fractions for analysis by LC-MS with ESI [84]. Detection in full scan mode over the *m*/*z* range 200–400 revealed the ergot biosynthetic intermediates chanoclavine-I and *N*-methyl-dimethylallyltryptophan. A similar method was applied to seeds and developing organs of morning glory plants (*Ipomoea*) [85]. The clavines chanoclavine lysergol, cycloclavine, and festuclavine were detected along with lysergic acid amides (ergine, lysergic acid α-hydroxyethylamide and ergonovine), and the ergopeptine ergobalansine. LC with high resolution tandem mass spectrometry (LC-HRMS/MS) was used to determine the major EA (lysergic acid amide, ergometrine, lysergol, elymoclavine, setoclavine, chanoclavine) and their stereoisomers in extracts of the seeds of the Hawaiian baby woodrose *Argyreia nervosa*, sold as a “legal high”. Seeds were powdered and extracted with methanol:water, centrifuged, filtered and analysed with comprehensive fragmentation patterns being described [86].

### 14.3. Forensic Analysis and Body Tissues

In forensic analysis LC–MS/MS was used for the quantitative determination of the dopamine agonist bromocriptine in human plasma [87]. Bromocriptine was extracted using mixed mode SPE cartridges. An aliquot of plasma containing an internal standard (α-ergocryptine) was loaded on to the conditioned SPE cartridge in 5% formic acid, and following rinses with 5% formic acid and methanol, the analyte was eluted with methanol containing ammonium hydroxide. The eluate solvent was evaporated and the bromocriptine dissolved in mobile phase for LC-MS/MS. The bromocriptine was detected in ESI mode using multiple selected reaction monitoring of the combined parent and product ions. The ^81^Br bromocriptine isotope had a less noisy signal than the ^79^Br isotope and so that corresponding mass ion was used. Sensitivity was greater with ESI than with APCI.

The extraction procedures for LSD and its simple relatives typically involve a single diethyl ether liquid–liquid extraction of a small volume (e.g., 1 mL) of blood or urine (adjusted to pH 9) prior to transfer of the analytes into the LC mobile phase [88]. LSD, 2-oxo-3-hydroxy-LSD, and *iso*-LSD (an impurity arising during synthesis) were similarly extracted from whole blood and urine with butyl acetate after adjusting to alkaline pH [57]. Toluene:dichloromethane (7:3) has been used to extract LSD, *iso*-LSD and *N*-desmethyl-LSD from basified urine for analysis by GC-MS/MS [48]. Blood was also extracted after clarification by addition of acetonitrile. The extracts were cleaned by cation exchange/mixed-mode SPE, the whole procedure having an extraction recovery of just over 50%.

A similar solvent extraction procedure with dichloromethane/diethyl ether (70:30) was used to determine LSD in 50 mg samples of hair [89]. LSD was extracted from blotter doses and from 50 mg hair samples in an ultrasonic bath with dimethylformamide and from plasma and urine with 1-chlorobutane before dilution in a solution containing the polarographic electrolyte tetrabutylammonium perchlorate and determination with anodic stripping voltammetry [90]. Liquid-liquid extraction with 1-chlorobutane was used to measure LSD in serum. The LSD was derivatised with MSTFA and detected by GC-MS [46].

EA extracted from cell cultures were determined using LC-FLD [14]. The mobile phase gradient used for the peptide alkaloids allowed to separate the ergometrine/ergometrinine pair. Stability tests in cell medium allowed the assignment of stability factors for each epimeric pair. Ergometrine and ergosine were the most resistant to epimerisation.

LC-FLD was used to measure ergosine, ergotamine and ergine in bovine serum based on C18 SPE trapping and clean-up [91]. Recovery of ergosine was 95% to 98% at the detection limit (0.5 ng/mL) and up to 24 ng/mL. A similar procedure was used for the determination of dihydroergotamine in serum, for which cyano (CN) SPE columns were used, with serum proteins being washed from the SPE columns with acetonitrile [61].

Reuschel *et al.* [16] extracted LSD and 2-oxo-3-hydroxy-LSD from urine using a cation exchange/mixed-mode SPE cartridge. Urine samples were added to the cartridge followed by a wash with dilute acetic acid and methanol, and elution with ethyl acetate containing aqueous ammonia solution. The analytes were derivatised with BSTFA and determined by GC-MS/MS in positive ion mode with selected ion monitoring. Lysergic acid methylpropylamide and 2-oxo-3-hydroxy-lysergic acid methylpropylamide were used as internal standards. The abundant protonated molecule ion of LSD.TMS (*m*/*z* 396) selected in the first quadrupole was subjected to collision-induced dissociation to form a product ion at *m*/*z* 295. The ions at *m*/*z* 295 were then mass selected in the third quadrupole. The protonated molecule ion of silylated 2-oxo-3-hydroxy-LSD and the 2-oxo-3-hydroxylysergic acid methylpropylamide internal standard (both *m*/*z* 500) formed abundant formed major product ions at *m*/*z* 309.

Neutral loss monitoring used to identify metabolites of LSD in urine revealed the presence of nor-LSD, 13 and 14-hydroxy-LSD, 2-oxo-3-hydroxy-LSD, trioxylated LSD, lysergic acid ethylamide and lysergic acid ethyl-2-hydroxyethylamide along with their glucuronide conjugates [88]. The similarly structured dopamine agonist bromocriptine has been determined with other compounds in rat plasma by LC-UV (wavelength λ max 240 nm) using a diode array detector [92] following protein precipitation with acetonitrile and liquid-liquid extraction with hexane:chloroform (70:30).

### 14.4. Pharmaceutical Formulations

Ergotamine tartrate is used to treat headache, migraine and related maladies. Methods for the determination of the drug in tablet preparations and production lines are ideally rapid and specific with low LOQs being less frequently required. Ergotamine tartrate has been determined in clinical preparations by a variety of methods including LC-UV, LC-FLD, CE. For body tissues LC-MS methods are generally required to achieve the low detection limits necessary. With LC-UV ergotamine can be detected with other active ingredients using a C18 column with a mobile phase of methanol-water-triethylamine (60 + 40 + 0.1, *v*/*v*/*v*) and detection at 254 nm [93].

Ergotamine was determined simultaneously with caffeine and metamizol in a solid headache formulation using a simple extraction with methanol/water 7:3 and separation by high-performance thin-layer chromatography (HPTLC) on silica plates [94]. Ergotamine was detected by fluorescence at 313/>340 nm, with other ingredients detected by UV. Ergotamine was confirmed by mass spectrometry in full scan ESI+ mode.

Ashour and Omar [95] validated a selective, sensitive and simple RP-HPLC method for the determination of ergotamine tartrate in pharmaceutical dosage. Following extraction with methanol the ergotamine tartrate was assayed using a C8 column with an isocratic methanol/formic acid solvent and UV detection. Bromocriptine mesylate was used as an internal standard.

CE with an alkaline phosphate buffer electrolyte in a 65 cm fused silica capillary was used to measure ergotamine tartrate in tablets [96]. Detection was performed at 210 nm. A pH of at least 9.8 was required to separate the tablets’ active ingredients however the total run time was high.

## 15. Conclusions

Analytical methods available so far have mainly focused on EA from *C. purpurea*. For other *Claviceps* spp. relevant to food and feed, data are sparse and no conclusions can be drawn. Recent developments in HPLC and HPLC-MS/MS technology (UHPLC) will most likely result in much higher throughput rates by reducing the sample preparation as well as separation time [19,40].

Many analyses are handicapped to a degree by the limited availability of standards, especially isotopically labelled standards useful for HPLC-MS/MS quantification. The availability of certified reference materials and regimes of interlaboratory trials are essential requirements in this area, which should be satisfied in the not too distant future.
